# Molecular Modelling and Dynamics Study of nsSNP in STXBP1 Gene in Early Infantile Epileptic Encephalopathy Disease

**DOI:** 10.1155/2019/4872101

**Published:** 2019-12-17

**Authors:** Krami Al Mehdi, Benhnini Fouad, Elkarhat Zouhair, Belkady Boutaina, Naasse Yassine, Ait El Cadi Chaimaa, Sifeddine Najat, Rouba Hassan, Roky Rachida, Barakat Abdelhamid, Nahili Halima

**Affiliations:** ^1^Laboratory of Genomics and Human Genetics, Institut Pasteur du Maroc, Casablanca 20360, Morocco; ^2^Laboratory of Physiology and Molecular Genetics, Department of Biology, Faculty of Sciences Ain Chock, B.P 5366 Maarif, Casablanca, Morocco; ^3^Laboratory of Cellular Signaling, Faculty of Sciences Meknes, Moulay Ismail University, Morocco

## Abstract

Early Infantile Epileptic Encephalopathy (known as Ohtahara Syndrome) is one of the most severe and earliest forms of epilepsy, characterized by early seizures onset. It affects newborns and children between two and six years old. Among the genes that have been associated with early infantile epileptic encephalopathy, the STXBP1 gene, which encodes the Syntaxin binding protein1a that is involved in SNARE complex formation, contributes to synaptic vesicles exocytosis. The aim of this study was to identify the most pathogenic polymorphisms of STXBP1 gene and determine their impact on the structure and stability of *Stxbp1 *protein. The high-risk nonsynonymous single nucleotide polymorphisms (nsSNPs) in the STXBP1 gene were predicted using 13 bioinformatics tools. The conservation analysis was realized by CONSURF web server. The analysis of the impact of the pathogenic SNPs on the structure of *Stxbp1 *protein was realized using YASARA software, and the molecular dynamics simulation was performed using GROMACS software. Out of 245 nsSNPs, we identified 11 (S42P, H103D R190W, R235G, D238E, L256P, P335S, C354Y, L365V, R406C, and G544D) as deleterious using in silico prediction tools. Conservation analysis results revealed that all these nsSNPs were located in conserved regions. The comparison of the hydrogen and hydrophobic interactions in the wild type *Stxbp1* structure and its mutant forms showed that all these nsSNPs affect the protein structure on different levels. The molecular dynamics simulations revealed that the total of nsSNPs affect the protein stability, residual fluctuation, and the compaction at different levels. This study provides helpful information on high risk nsSNPs that may affect the *Stxbp1* protein structure and function. Thus, these variants should be taken into consideration during the genetic screening of patients suffering from early infantile epileptic encephalopathy.

## 1. Introduction

Epilepsy is a cerebral disorder defined by repetitive recurrent or spontaneous epileptic seizures, due to an imbalance between the excitatory and inhibitory mechanism of the nervous system [1]. According to the World Health Organization, more than 50 million people suffer from this disease worldwide, accounting for 0.6% of the global morbidity.

There are different types of epilepsy based on the clinical description, the electroencephalogram results, and the age of onset. Thus, different epileptic forms can be diagnosed including Early Infantile Epileptic Encephalopathy (EIEE) [2].

Early infant epileptic encephalopathy, also recognized as Ohtahara syndrome, is a neonatal age-dependent neurological disorder, which was first described by Ohtahara in 1976 as a devastating disease that affects neonates/infants, hence its name [3]. This rare form of epilepsy is characterized by a preferential early age of onset, tonic seizures, and infantile spasms within the first 3 months of life in most cases leading to a deregulation of brain functions and apparent abnormalities on the electroencephalogram [4]. This clinical entity includes two syndromes: the first, named West syndrome (also known as infantile spasm or generalized reflection epilepsy) is known as a rare form of epilepsy that affects 3–12  month-old infants and is characterized by the occurrence of spasms, accompanied by a progressive decline in neurocognitive functioning and development. This syndrome is generally due to a cerebral anomaly (brain malformations, brain lesions, etc.) or genetic abnormalities (trisomy 21, mutation of the ARX or STK9 gene) [5, 6]; The second, called Lennox-gastant syndrome is a severe form of epileptic encephalopathy that affects 2 to 6 year-old children, this condition is characterized by psychomotor retardation accompanied by different types of frequent crises (tonic, axial, diurnal and nocturnal crises, etc.) [7].

Several causes may interfere with the early infantile epileptic encephalopathy development including structural brain abnormalities as well as other genetic factors involving variants of the KCNQ2, ARX, CDKL5, and STXBP1 genes [8, 9].

The STXBP1 (also known as Munc18) is a gene located on the long arm of chromosome 9 at position 34.11 [10], and composed of 20 exons [11], which encodes the Syntaxin1a binding protein (*Stxbp1*), a protein of the SEC1 family. *Stxbp1* protein is made of 603 amino acids distributed over 3 domains [12]. The first domain comprises a peptidic sequence from the 4th to the 134th residue, which consists of a five-stranded parallel *β*-sheet flanked by five *α*-helixes [12]. The second domain which contains residues from positions 135 to 245 and 480 to 592 adopts a six-stranded *β*-sheet with five parallel and one antiparallel structure. The third domain which is composed of residues 246 to 479, it is subdivided into 2 sub-domains: 3a and 3b [12].

The *Stxbp1* is abundantly expressed in the brain and is suspected to be involved in synaptic vesicle exocytosis [13]. Indeed, the release of neurotransmitters in the synaptic space requires the regulated fusion of the synaptic vesicle with the plasma membrane; this mechanism is called the docking and priming of vesicles [14]. Among the most important proteins involved in this process is the synaptic SNARE complex (Soluble N-éthylmaleimide-sensitive-factor Attachment protein REceptor). This complex is composed of the Synaptobrevin protein of the synaptic vesicle, and the presynaptic membrane proteins SNAP25 and Syntaxines1a. These 3 proteins form a helical bundle creating a bond between the synaptic vesicle and the presynaptic membrane [15]. *Stxbp1* is crucial to the SNARE complex formation after establishing the connection with Syntaxines1a by promoting the change of its conformation [16].

Mutations affecting the STXBP1 gene lead to a nonfunctional protein unable to bind the syntaxin1a, leaving it inactive and unable to bind in its turn the Synaptobrevin and synaptosomal-associated protein 25 (*SNAP25*) proteins. The absence of SNARE complex will no longer allow the binding of the synaptic vesicle to the presynaptic membrane, thus, blocking the secretion of inhibiting neurotransmitters and causing a hyper excitation of the neurons, leading consequently to triggering of a convulsion attack [17].

In order to highlight the importance of the STXBP1 gene in exocytosis, an ultrastructural analysis was performed by Weimer and his collaborators on presynaptic terminations in mutant protein. They have demonstrated that synaptic vesicles are more dominant and less associated with the plasma membrane [18]. In addition, to determine the role of STXBP1 in Early Infantile Epileptic Encephalopathy, Saitsu et al. have identified four heterozygous missense mutations: V48D (Exon 5), C180Y (Exon 7), M443R (Exon 15), and G544D (Exon18) inducing the disease in the cases of one woman and three men [9]. Recently, missenses mutations have been identified in several studies, including the R292H, M262T, and C354Y in three patients with encephalopathy associated with awake bruxism [19]. Furthermore, the M252T mutation has been identified in a man with clonic seizures and epileptic spasms [20], while the R551C mutation has been reported in Stamberger's systematic review [21].

Bioinformatics is increasingly becoming a prominent part of several areas of biology essentially implicating molecular biology, computer science, statistics, and genetics which play a crucial role in the analysis of gene and protein expression and regulation [22]. The computational tools help to understand the genomic variation implication particularly the nsSNPs by predicting their impact on amino acid residues change in proteins. In other words, nsSNPs' prediction tools are executed to predict the potential structural and functional impact caused by these variants [23]. In order to evaluate more precisely the structural impact caused by amino acid changes, a molecular dynamics simulation can be performed, allowing observations of changes in many parameters such as stability and flexibility of the protein [24].

The aim of this study is to identify the most deleterious nsSNPs and evaluate their pathogenic impact on the protein structure, using molecular modeling and molecular dynamics simulation.

## 2. Data and Methods

### 2.1. Data Source

Literature search on the STXBP1 gene was conducted using PubMed, Science Direct, and Online Mendelian Inheritance in Man (OMIM) databases, opting only for publications that prove the functional association of STXBP1 gene mutations with early infantile epileptic encephalopathy. Our research strategy was based on the keywords (Human and STXBP1) and (Early Infantile Epileptic Encephalopathy and epileptic disease).

The amino acid sequence of *Stxbp1 *was obtained from the Uniprot database (https://www.uniprot.org) [P61764].

The three-dimensional structure of the native protein *Stxbp1* server. The model adopted to form the three-dimensional standard was chosen based on the sequence identification and the QMEAN function [2525–29]. The three-dimensional structures of mutant forms were generated using the same server in order to minimize all structures by GROMACS 5.1.4 program [30].

SNPs for STXBP1were provided by the National Center for Biotechnology Information (https://www.ncbi.nlm.nih.gov/snp/?term=STXBP1) and the Ensembl database https://www.ensembl.org/Homo_sapiens/Gene/Variation_Gene/Table?db=core;g=ENSG00000136854;r=9:127579370-127696027).

### 2.2. Analysis and Identification of the Most Damaging SNPs

To determine the impact of SNPs on the structure of *Stxbp1*, thirteen computational tools were applied (Table 1). These algorithmic analyses include: SIFT [31], POLYPHEN [32], Provean [33], Mcap [34], SNAP [35], Phd-SNP [36], MAPP [37], Condel [38], LRT [39], Mutation assaror [40], Mutation Taster [41], PANTHER [42] and PredictSNP [43]. Only the deleterious SNPs predicted by eight algorithms were studied.

### 2.3. Conservation Analysis

The variable and conserved regions of STXBP1 were characterized using the Consurf web server (https://consurf.tau.ac.il/). Based on homology, this algorithm estimates the degree of conservation of amino acid sites [44].

### 2.4. Impact of Deleterious SNPs on Hydrogen and Hydrophobic Interactions of Stxbp1

The analysis of the difference in the hydrogen and hydrophobic bonds between the amino acids of the wild-type protein and its mutant forms were performed by using YASARA software [45].

### 2.5. Molecular Dynamics Simulation

The simulation of the WT-*Stxbp1* structure and its variants by molecular dynamics was performed using the GROMACS 5.1.4 software package with the CHARMM 27 force field [46].

The protein atoms were placed in a cubical box and other periodic boundary conditions were optimized to perform the simulations. To solvate and neutralize the system, sodium ions were added. Energy minimization was executed using steep descent method of 5000 steps to have a stable conformation.

After minimization, Canonical Ensembles (NVT) and isobar isothermal Ensembles (NPT) were performed, respectively, with a constant temperature of 300 K for 100 ps for NVT followed by a constant temperature of 300 K and a constant pressure of 1 atm per 100 ps for NPT.

Molecular dynamics simulation was performed at 300 k for 5000 ps. The root-mean-square-deviation (RMSD), root-mean-square-fluctuation (RMSF), and radius of gyration (Rg), were calculated by g-rmsd, g-rmsf and g-Rg, respectively [47]. The resulting graphics for these parameters were designed using the QtGrace.22 program.

## 3. Results

### 3.1. Distribution of SNPs

**Figure 1:**
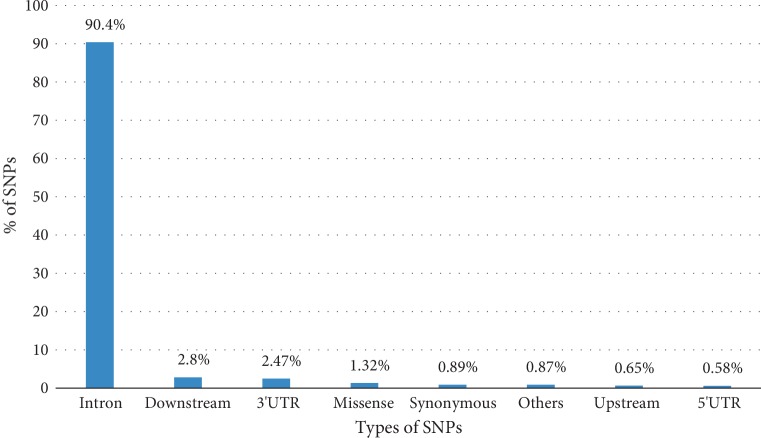
Distribution of SNPs present in the STXBP1 gene.

The set of STXBP1 SNPs retrieved from the dbSNP database reached a total of 18 534 SNPs. the distribution of SNPs is illustrated in Figure 1. The majority of SNPs are localized in the intronic part, whereas the missenses variants present in the coding part represent 1.32%.

### 3.2. The Most Deleterious SNPs Identified in STXBP1

A variety of computational tools were used to predict the pathogenic effect of nonsynonymous SNPs on the protein structure. The thirteen applied algorithms were: SIFT, Polyphen, Provean, Mcap, SNAP, Phd-SNP, MAPP, Condel, LRT, Mutation assessor, Mutation Taster, PANTHER, and PredictSNP (Table 1).

Out of 245 nsSNPs, only 11 were predicted as deleterious by at least nine tools. Most algorithms successfully predicted that the S42P, H103D, L256P, and C354Y variants were deleterious, while, eight algorithms prognosticated that the L365V variant was deleterious. The four mutations: R190W, R235G, D238E, and R406C were predicted to be damaging by eleven algorithms, whereas, ten other algorithms suggested that both mutations P335S and G544D were deleterious (Table 2).

### 3.3. Conservation Analysis

Human health is prone to be affected by many variations. Thus, the identification of these requires some evolutionary information. In order to predict the conservation frequency of *Stxbp1* residues, an analysis using the Consurf web server was performed. The analysis results showed that 11 nsSNPs were located in highly conserved regions.

**Figure 2:**
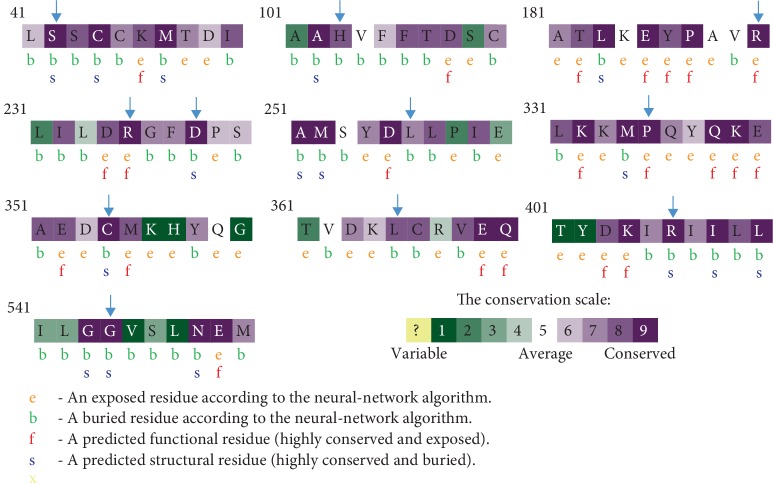
Evolutionary conservation of amino acids in Syntaxin binding protein1a analysed by Consurf.

The results predicted that R190, R235, and P335 were functional and exposed residues. We also educed that S42, D238, C354, R406, and G544 were buried and structural. The variants H103, L256, and L365 were predicted as buried residues (Figure 2, Table 3).

### 3.4. Structural Modeling

The PDB server generated three models for the *Stxbp1* complex linked to Syntaxin 1A (3PUJ, 4JEH and 4JEU) [48]. Indeed, the crystallized model (4JEU) with a resolution of 3.2 Å proved to be the best choice for this study, while the syntaxin1A protein was eliminated using YASARA software.

The eleven nsSNPs predicted as deleterious were individually substituted at the FASTA sequence level of the native protein.

These were submitted to the Swiss Model homology modeling tool. The energy minimization was achieved by the GROMACS software. The resulting structures were visualized by YASARA.

### 3.5. Comparison of Native and Variants Structures of Stxbp1

**Figure 3:**
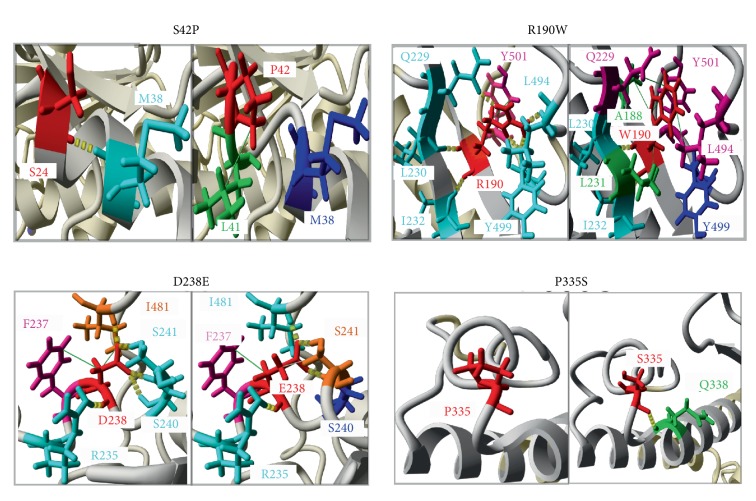
Example of the comparison of the native STXBP1 protein structure and its mutant forms. S42 (wild type-STXBP1) and 42P (variant protein), R190 (wild type-STXBP1) and 190 W (variant protein), D238 (wild type-STXBP1) and 238E (variant protein) and P335 (wild type-STXBP1) and 335S (variant protein). Residues substituted are showed in red, residues involved in hydrogen bonds are marked in cyan, residues participate in hydrophobic interactions are indicated in magenta, residues participate in both hydrogen bonds and hydrophobic interactions are marked in orange, and the residues which lost a hydrogen bonds and/or hydrophobic interactions are marked in blue, and the new residues appeared are indicated in green. Hydrogen bonding is marked by yellow dashed lines and hydrophobic interactions are shown by green lines.

The 11 polymorphisms already predicted as deleterious by the thirteen prediction software revealed that the structural protein changes in comparison with the native protein by the YASARA software. The hydrogen and hydrophobic interactions change results of STXBP1 protein are represented in Table 4. Furthermore, the Figure 3 shows an example of YASARA results of the four variants S42P, R190W, D238E, and P335S.

### 3.6. Molecular Dynamics Simulation

The evaluation of the impact of pathogenic SNPs on the protein structure of *Stxbp1* was analyzed by simulations in molecular dynamics using GROMACS 5.1.4.

After generating the three-dimensional structures of the wild-type protein and its mutated forms, an analysis of the molecular dynamics simulation trajectories for 5000 ps was performed using RMSD, RMSF and Rg.

#### 3.6.1. Stability Analysis

**Figure 4:**
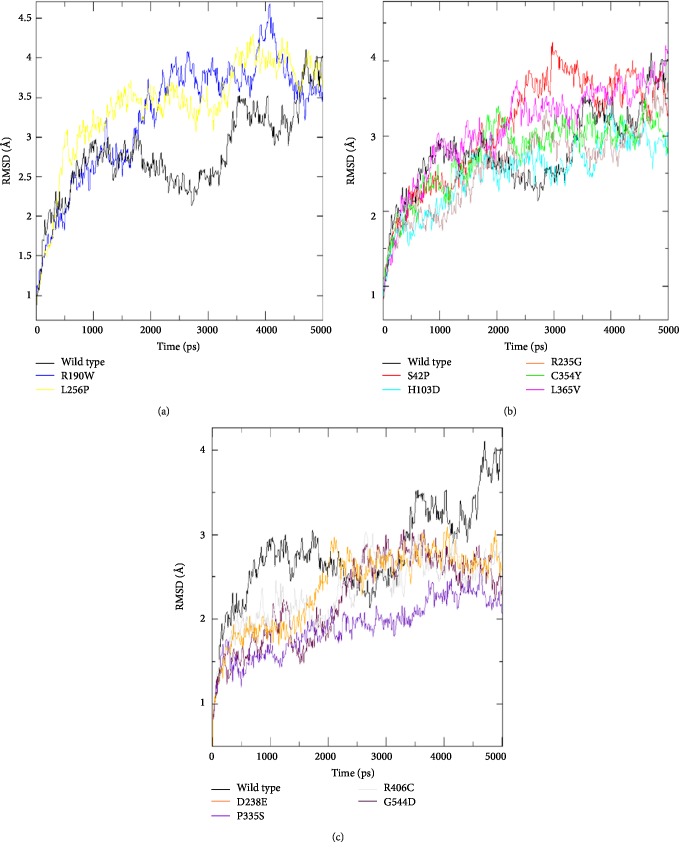
Backbone Root Mean Square Deviation (RMSD) of STXBP1 native protein and mutant forms for 5000 ps of molecular dynamics simulation. Color scheme: (a) wt-STXBP1 (black), R190W (blue), and L256P (yellow), (b) wt-STXBP1 (black), S42P (red), H103D (cyan), C365Y (green), L365V (magenta) and R235G (brown), (c) wt-STXBP1 (black), D238E (orange), P335S (violet), G544D (maroon), and R406C (grey).

At the beginning of the dynamics simulation, the RMSD value of WT-*Stxbp1* was in the order of 0.05 Å. This value ranged between 2.8 Å to 2.4 Å during the first to the third nanosecond, reaching 4.1 Å during the last period of this simulation. However, Each *Stxbp1* variant had a particular RMSD trajectory (Figure 4).

For the protein with the variant P335S and G544D, its RMSD trajectory showed a lower value compared to the wild-type protein during the simulation.

Concerning the variant D238E and R406C, their RMSD values revealed a decrease throughout the first 2200ps and also after the 3300ps, whereas between these two intervals, the two trajectories showed no significant difference.

For the R190W variant, no difference was observed during the first 1700 ps. However, the trajectory of R190W showed a significant increase of the RMSD value compared to the native protein, between 1700ps and 4500ps.

Regarding the L256P variant, its RMSD trajectory indicated a value higher than the native protein, during the period of 500 to 4500ps.

The S42P, H103D, R235G, C354Y, and L365V variants showed a trajectory generally similar to the native protein one during dynamics simulation.

The RMSD analysis indicated that the variants D238E, P335S, R406C, and G544D increased the stability of the protein while the variants R190W and L256P reduced it.

#### 3.6.2. Flexibility Analysis

**Figure 5:**
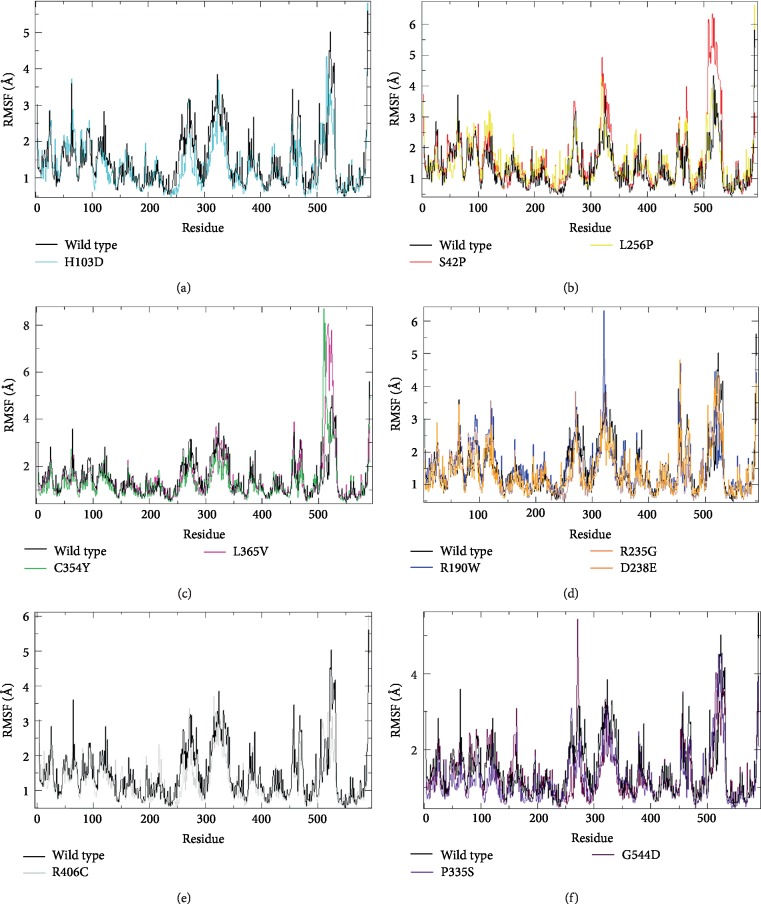
Backbone Root Mean Square Fluctuation (RMSF) of the wild type STXBP1 protein and its mutant forms for 5000ps of molecular dynamics simulation. Color scheme: (a) a wt-STXBP1 (black) and H103D (cyan), (b) wt-STXBP1 (black), S42P (red) and L256P (yellow), (c) wt-STXBP1 (black), C354Y (green) and L365V (magenta), (d) wt-STXBP1 (black), R190W (blue), R235G (brown) and D238E (orange), (e) wt-STXBP1 (black) and R406C (grey), (f) wt-STXBP1 (black), P335S (violet) and G544D (maroon).

The difference in flexibility between amino acids was deduced by RMSF analysis during molecular dynamics simulation (Figure 5).

The flexibility of the native *Stxbp1* was presented by values between 0.49 Å and 5.6 Å, with 0.75 Å to 3.5 Å for the domain 1, 0.49 Å to 5.6 Å for the domain 2 and 0.63 Å to 3.8 Å for the domain 3.

Concerning the RMSF values of the domain 2 of the *Stxbp1*, the variants L365V, C354Y, L256P, and S42P displayed values higher than the native protein and influences the flexibility by its increase, while the variants R190W, R235G, D238E, P335S, and G544D reduce the flexibility since they showed lower values.

In addition, a difference in the domain 3 of the STXBP1 gene was observed in S42P, R190W, L256P, and D238E, and G544D; these variants showed an increase in the RMSF values in comparison with the wild-type protein, which augment the flexibility. Furthermore, the variants P335S and C354Y indicated a decrease of RMSF values compared to wt-*Stxbp1*, allowing the flexibility reduce. Note that no significant difference was reported for H103D, L365V, and R406C mutants.

For the domain 1, a decrease was observed in the RMSF values of the P335S, C354Y, L365V, R406C, and G544D mutants, compared with the native protein. Thus, it was reflected by the reduction in the flexibility, while for the other variants, there was no significant difference.

#### 3.6.3. Gyration Analysis

**Figure 6:**
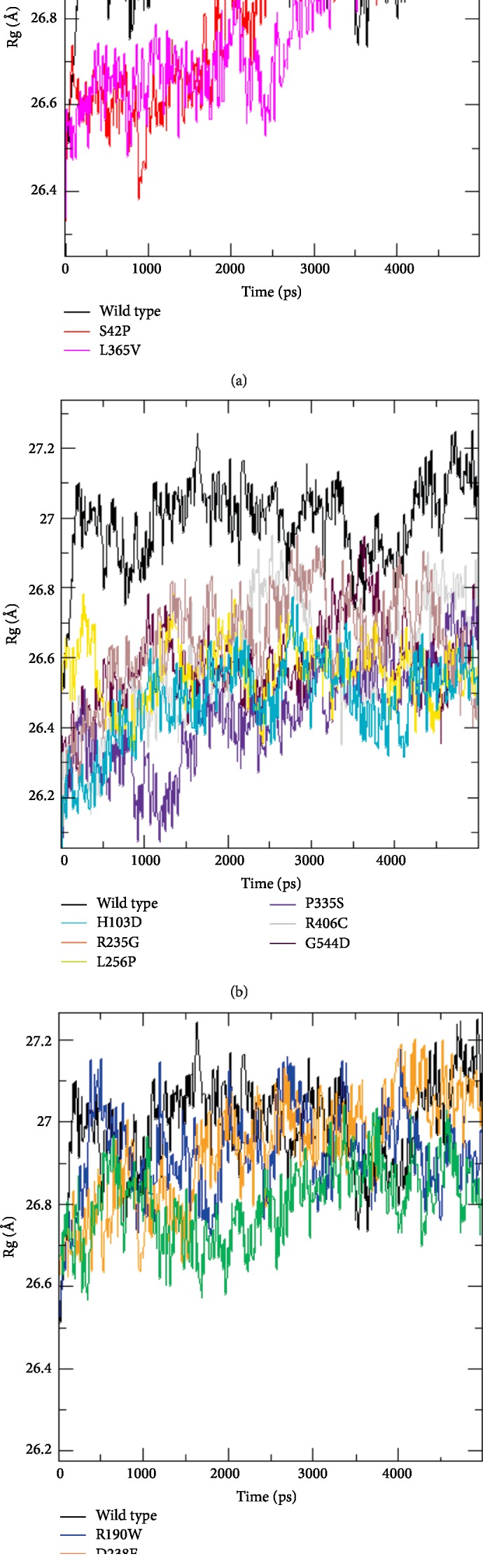
Radius of gyration for STXBP1 protein and its variants proteins for 5000 ps of molecular dynamic simulation. Color scheme: (a) wt-STXBP1 (black), S42P (red), and (L365V) magenta. (b) wt-STXBP1 (black), H103D (cyan), R235G (brown), L256P (yellow), R406C (grey), P335S (violet) and G544D (maroon). (c) wt-PIWIL1 (black), (R190W) blue, (D238E) orange, and (C354Y) green.

Parameters such as overall dimensions and the compaction level of the molecules were evaluated by a radius of gyration analysis (Figure 6).

At the beginning of the simulation, the Rg value of the native protein was about 26.3 Å. During the simulation, this value varied between 26.5 Å and 27.3 Å, to reach a value of 27.1 Å towards the end.

The Rg values of the H103D, R235G, L256P, P335S, R406C, and G544D variants were significantly lower than that of the native protein. As for the S42P and L365V variants, their Rg values were lower than the native protein values during the first 2000 ps. After this period, the three trajectories showed no significant differences. Furthermore, the Rg values of the R190W, D238E, and C354Y showed a trajectory generally similar to the native protein during this simulation.

The Rg analysis stated that proteins with the variants S42P, H103D, R235G, P335S, L365V, R406C, and G544D had a higher level of compaction than the wild-type protein.

## 4. Discussion

The process of identifying nucleotidic variations in genes and their effect on the corresponding protein in vitro by conventional techniques is not only difficult but also too slow and laborious to implement. In order to ease this, it is useful to execute studies using computational biology [49]. Single nucleotide polymorphism (SNP) is a single nucleotide variation which is the basis of genetic variation [50]. The majority of SNPs are neutral. However, some so-called nonsynonymous SNPs (nsSNP) can affect a gene, causing human predisposition to several diseases.

Since its identification, the syntaxin-binding proteins 1 play an essential part in the regulation of synaptic vesicle docking and fusion [10, 13]. Furthermore, a large number of studies have revealed the association of the STXBP1 gene mutations in the Early Infantile Epileptic Encephalopathy patients [9, 19, 20].

This computational study aims to analyze the effect of nonsynonymous SNPs on the structure and functioning of the protein through prediction tools. nsSNPs were collected from the dbSNP database. These were subjected to thirteen prediction algorithms. This analysis was able to identify 11 variants (S42P, H103D R190W, R235G, D238E, L256P, P335S, C354Y, L365V, R406C, and G544D) as the most deleterious nsSNPs of the STXBP1 gene by at least 9 computational tools. The difference between the results of the prediction software used in this study revealed the importance of using more than one algorithm to estimate the effect of variations on the structure and function of the protein [51].

Conservation analysis results showed that all these nsSNPs were located in conserved regions. Indeed, According to Miller and Kumar, the highly conserved amino acids are located in biologically active sites. When these residues are substituted, the biological activities are affected [52].

The eleven SNPs predicted as pathogens showed a loss of hydrogen and hydrophobic bonds compared to the wild-type protein after visualization by YASARA software. Indeed, Wang and Moult have shown that approximately 80% of their disease related to nsSNPs produced a destabilization protein [53]. It should be noted that the S42P/H103 and L256P/P335S variants were located respectively in domains 1 and 3a, and formed the central cavity that allows the binding to Syntaxin 1a. These variants may induce a change in spatial conformation, and consequently the destabilization of the “Syntaxin 1a-*Stxbp1*” complex, which may be a favorable EEIP risk factor. The loss or gain of hydrogen bonds, hydrophobic interactions, and salt bridges due to deleterious SNPs can affect the normal protein structure and function [54, 55].

Currently, bioinformatics approaches use simulation to elucidate the various impacts of mutations on proteins [56]. The real behaviour of molecules in their environment is reproduced by molecular dynamics simulations [57]. This computational platform provides more detailed information regarding the particle motion, stability, flexibility and overall dimensions of the protein as a function of time [58]. Moreover, this powerful analysis presents the best correlation with experimental studies [59]. In fact, these properties are interdependent; they must be examined together when studying protein structure, while the variants that inhibit one property of a protein may directly influence the other property [60, 61].

In our study, RMSD analysis showed that the mutated proteins with the variants D238E, P335S, R406C, and G544D increased its stability, whereas, the two variants R190W and L256P reduced it. Theses deleterious SNPs might induce a maximum damage on the protein stability 1. Indeed, according to Yue and Moult, 25% of pathogenic SNPs might affect the protein function in the human population by the protein stability alteration [62]. Moreover, some studies have shown the increase in degradation, aggregation, and misfolding of the protein is the result of reduced stability [63, 64]. The variants R190W, L256P, L365, and R406C induced the protein stability change. Indeed, some studies of the evolutionary stability and mutations that affect protein-coding genes have demonstrated that leucine, serine, and arginine are the major amino acids that affect protein stability in mutants [65].

In our study, the analyses of RMSF showed that the 11 nsSNPs influence the flexibility at different levels. The flexibility is a crucial property in protein activity. It allows the proteins to reply to environmental changes and chemical modifications. The molecular flexibility controls several functions such as enzymatic catalysis and regulation of protein activity. Indeed, a disruption that affects the proteins flexibility may potentially interfere with their function accordingly disease development [66–68].

The Rg describes the overall spread of the molecule and is measured as the root mean square distance of atoms collection from their common centre of gravity. Indeed, Lobanov et al. have demonstrate that the Radius of Gyration is an Indicator of Protein Structure Compactness [69, 70]. In the current study, the Rg analysis revealed that the variants S42P, H103D, R235G, P335S, L365V, R406C and G544D had a higher level of compaction than the wild-type protein.

Among our results we have found three pathogenic variants which were proved to be involved in the etiology of the disease, according to the following studies. Carvill et al. collaborators conducted a study based on high-rate resequencing of 65 known genes in 500 patients, with the goal of identifying new pathogenic variants involved in epilepsy and studying the phenotypic spectra of these genes. This study was able to identify the arginine substitution at position 190 by the tryptophan amino acid (R190W) at the STXBP1 gene in patients with early epileptic encephalopathy [71].

In addition, another study by Parrini et al. targeted 349 patients with an epilepsy that began in the first years of their lives. This team could detect a substitution of arginine by cysteine at position 406 of the STXBP1 gene using a sequencing of panel of 30 genes in a patient with Neonatal Epileptic Encephalopathy [72]. The substitution of the Glycine amino acid by a negatively charged residue such as aspartic acid at position 544 had already been identified by Saitsu et al. collaborators as damaging for the *Stxbp1*. This study was performed on 14 Japanese citizens with early infantile epileptic encephalopathy.

The G544D mutation was observed in one patient who had been diagnosed with several symptoms including tonic seizures after ten days of birth, delayed development, and spastic diplegia [9].

## 5. Conclusion

In this study, the screening analysis using a variety of bioinformatic tools revealed that the structure and/or function of STXBP1 protein can be perturbed by deleterious nsSNPs.

The detailed analysis of the findings of our study underlined the importance of all the variations, in particular at the 42th, 103th, 256th, and 335th positions located in both domains 1 and 3a involved in syntaxin1a binding and which may be at the origin of the destabilization of the *Stxbp1* -syntaxin complex and consequently of the occurrence of the disease. Indeed, the whole STXBP1 gene will have to undergo a structural and functional analyses to reveal any potential implication in early infantile epileptic encephalopathy.

Furthermore, functional analyses are needed to elucidate the biological mechanisms of these polymorphisms in the early infantile epileptic encephalopathy.

## Figures and Tables

**Table 1:** List of prediction tools and their URL.

Prediction tools	URL	References
Sift	https://sift-dna.org	Sim et al. [31]
Plyphen	https://genetics.bwh.harvard.edu/pph2/	Adzhubei et al. [32]
Mutation assessor	https://mutationassessor.org/r3/	Reva et al. [40]
Mutation taster	https://www.mutationtaster.org/	Schwarz et al. [41]
PROVEAN	https://provean.jcvi.org/index.php	Choi et al. [33]
Condel	https://bbglab.irbbarcelona.org/fannsdb/help/condel.html	González-Pérez et al. [38]
M-CAP	https://bejerano.stanford.edu/mcap	Jagadeesh et al. [34]
LRT	https://www.genetics.wustl.edu/jflab/lrt_query.html	Chun et al. [39]
Predict SNP	https://loschmidt.chemi.muni.cz/predictsnp/	Bendl et al. [43]
MAPP	https://mendel.stanford.edu/SidowLab/downloads/MAPP/index.html	Stone and Sidow [37]
PhD-SNP	https://snps.biofold.org/phd-snp/phd-snp.html	Capriotti, Calabrese, and Casadio [36]
SNAP	https://www.bio-sof.com/snap	Johnson et al. [35]
PANTHER	https://www.pantherdb.org/tools/	Mi et al. [44]

**Table 2:** Results of the deleterious STXBP1 gene SNPs predicted by 13 prediction tools.

SNP id	Substitution	Frequency	SIFT	PolyPhen	Mutation assessor	Mutation Taster	PROVEAN	Condel	M-CAP	LRT	Predict SNP	MAPP	PhD-SNP	SNAP	PANTHER
rs886041668	S42P	-	D	PD	M	D	D	D	D	D	D	D	D	D	D
rs1230238574	H103D	0,00001 (1/125568, topmed)	D	PD	M	D	D	D	D	D	D	D	D	D	D
rs796053355	R190W	-	D	PD	M	D	D	D	D	D	D	D	D	D	-
rs796053359	R235G	-	D	PD	M	D	D	D	D	D	D	D	D	D	-
rs587784456	D238E	-	D	PD	M	D	D	D	D	D	D	D	D	D	-
rs1057524834	L256P	-	D	PD	M	D	D	D	D	D	D	D	D	D	D
rs1085307916	P335S	-	D	PD	M	D	D	D	D	D	D	N	D	N	D
rs796053365	C354Y	-	D	PD	M	D	D	D	D	D	D	D	D	D	D
rs796053358	L365V	0,00000 (1/246266, GenomAD)	D	PD	L	D	D	D	D	D	N	N	N	N	D
rs796053367	R406C	-	D	PD	M	D	D	D	D	D	D	D	D	D	-
rs121918317	G544D	-	D	PD	M	N	D	D	D	D	D	D	D	D	-

N: Neutral; D: Deleterious; PD: probably damaging; M: medium; L: low; -: unknown.

**Table 3:** Consurf result.

Amino acid position	Consurf result		
	Conservation score	Localisation of residue	Role of residue
S42	9	Buried	Structural residue
H103	8	Buried	-
R190	9	Exposed	Functional residue
R235	9	Exposed	Functional residue
D238	9	Buried	Structural residue
L256	7	Buried	-
P335	9	Exposed	Functional residue
C354	9	Buried	Structural residue
L365	8	Buried	-
R406	9	Buried	Structural residue
G544	9	Buried	Structural residue

**Table 4:** Effect of nsSNPs of STXBP1 gene on Hydrogenic and hydrphobic interactions.

SNP ID	Substitution	Variation impact on the hydrogen bond	Variation impact on hydrophobic bond
rs886041668	S42P	Loss of hydrogen bond with M38 residue	Gain of hydrophobic bond with L41 residue
rs1230238574	H103D	Loss of hydrogen bond with E72 residue	Loss of hydrophobic bond with T129 residue and gain of hydrophobic bond with F105 residue
rs796053355	R190W	Loss of hydrogen bonds with Q229, L494 and Y499 residues	Gain of hydrophobic bonds with A188, Q129, L231 and L494 residues
rs796053359	R235G	Loss of hydrogen bonds with Y157, D238, G544 and E549 residues	Loss of hydrophobic bonds with L446 and L573 residues and gain of hydrophobic bonds with L542 residue
rs587784456	D238E	Loss of hydrogen bonds with S240 and S241 residues	Loss of hydrophobic bond with I481 residue, gain of hydrophobic bond with S241 residue
rs1057524834	L256P	Loss of hydrogen bond with M252 residue	Loss of hydrophobic bonds with V362, M252 residues
rs1085307916	P335S	Gain of hydrogen bond with Q338 residue	No change
rs796053365	C354Y	Y354 residue has kept the same hydrogen bonds with L250, Y358 residues	Gain of hydrophobic bonds with F249, S253, L257, I296, and L350 residues
rs796053358	L365V	No change	Loss of hydrophobic bonds with T361, I393, and V399 residues and gain of hydrophobic bond with E369 residue
rs796053367	R406C	Loss of hydrogen bonds with V243 and D255 residues	No change
rs121918317	G544D	Gain of hydrogen bond with V573	Loss of hydrophobic bonds with S569 and H571 residues
